# Structures of *Mycobacterium tuberculosis* isoprenyl diphosphate synthase Rv2173 in substrate-bound forms

**DOI:** 10.1107/S2053230X25002298

**Published:** 2025-04-01

**Authors:** James A. Titterington, Ngoc Anh Thu Ho, Charles P. H. Beasley, Francis Mann, Edward N. Baker, Timothy M. Allison, Jodie M. Johnston

**Affiliations:** a Biomolecular Interaction Centre and School of Physical and Chemical Sciences, University of Canterbury. Christchurch, New Zealand; b School of Biological Sciences University of Auckland Auckland New Zealand; c SC Johnson & Son 1525 Howe Street Racine WI53403 USA; Centre for Cellular and Molecular Biology, Hyderabad, India

**Keywords:** *Mycobacterium tuberculosis*, isoprenyl diphosphate synthases, Rv2173, dimethylallyl diphosphate, isoprenyl diphosphate

## Abstract

We report structures of the *Mycobacterium tuberculosis* isoprenyl diphosphate synthase Rv2173 in three forms: apo and two substrate-bound forms [isoprenyl diphosphate (IPP) and dimethylallyl diphosphate (DMAPP)]. The protein possesses a canonical all-α-helical *trans*-isoprenyl diphosphate synthase fold that is dimeric in each form. There are some differences between the structures: the IPP-bound form shows IPP bound in the DMAPP/allylic substrate-binding site with three divalent metal ions bound around the IPP and the complete C-terminus closing around the active site, while the apo and DMAPP-bound forms are more open, with some of the C-terminal region disordered, supporting suggestions that the C-terminus is important in substrate entry/product exit. In the DMAPP form DMAPP occupies the expected allylic substrate site, but only two metal ions are associated with the binding, with the DMAPP diphosphates adopting a slightly different binding pose compared with IPP in the same site, and the third metal-binding site is unoccupied. In no case is the IPP binding site occupied by IPP. There has been some uncertainty regarding product length for Rv2173, with variable lengths being reported. In the structures reported here, the ‘capping’ residue at the bottom of the binding cavity is tryptophan and comparison with other IPP synthases suggests that the structure of Rv2173 is most consistent with a C_10_–C_15_ final product size.

## Abstract 2

We report the structure of *M. tuberculosis* isoprenyl diphosphate synthase Rv2173 in three forms, including two with substrate (isoprenyl diphosphate and dimethylallyl diphosphate) occupying the allylic substrate site in different binding poses, with different numbers of metal ions bound. The homodimeric structures possess a canonical all-α-helical *trans*-isoprenyl diphosphate synthase fold, which supports small but significant differences, notably in the ordering of the C-terminus that closes the active site.

## 1. Introduction

**Figure 1:**
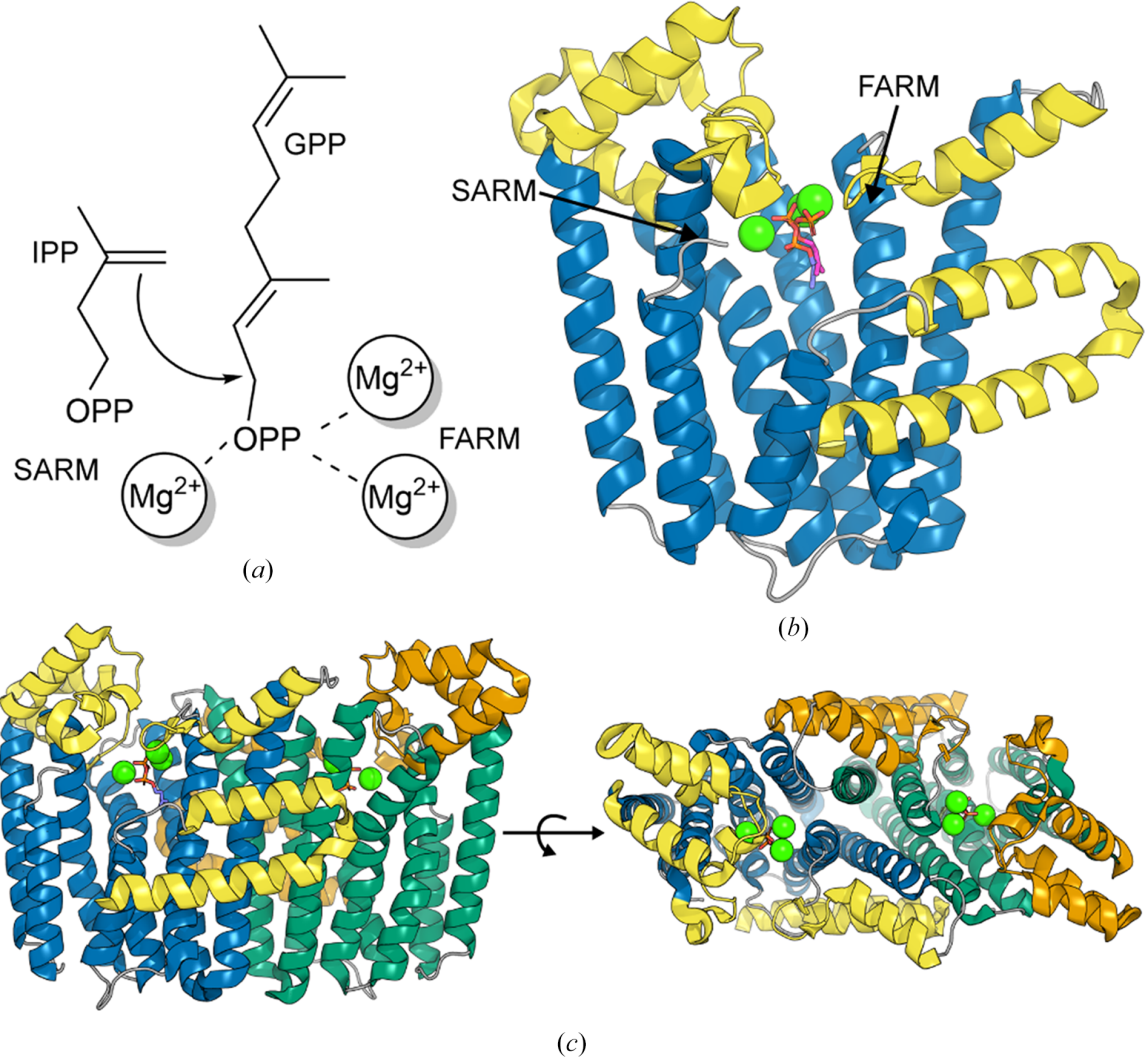
(*a*) IPP synthase reaction. (*b*) Rv2173 monomer (IPP-bound structure) with the core eight-helix bundle coloured blue and the additional helices in yellow. (*c*) Side (left) and top-down (right) views of the homodimer. Monomer 1 is coloured as in (*b*) and monomer 2 is in green with additional helices in orange. The metals from the IPP structure are shown as green spheres and the substrates IPP and DMAPP (from the superimposed DMAPP-bound structure) are shown as purple and pink sticks, respectively.

Polyisoprenyl diphosphates provide polyisoprenyl building blocks used in the biosynthesis of isoprenoid compounds such as steroids, chlorophyll, carotenoids and monoterpenes/diterpenes (Wang & Ohnuma, 2000 ▸). These compounds have a wide range of lengths and varying stereochemistry at the double bonds (*E*/*Z* and all-*E*). Polyisoprenyl diphosphates are made via the sequential condensation of five-carbon iso­pentenyl diphosphate (IPP) units. The enzymes responsible for this condensation are known as prenyltransferases or isoprenyl diphosphate synthases (Ohnuma *et al.*, 1996 ▸). Initial condensation involves the addition of IPP to its isomer dimethylallyldiphosphate (DMAPP) to form a product that can be further elongated by further condensation with additional IPP molecules (Ohnuma *et al.*, 1996 ▸; Fig. 1 ▸*a*). For the all-*E* enzymes this is believed to occur via a sequential ionization–condensation–elimination mechanism (Liang, 2009 ▸). The allylic substrate (for example DMAPP or longer allylic substrates) is ionized to form a resonance-stabilized allylic cation, which is then attacked by the double bond of IPP. Elimination/double-bond formation of this condensed species is then mediated by proton abstraction by the diphosphate group formed during allylic substrate cleavage (Liang, 2009 ▸; Christianson, 2017 ▸). This can occur over several rounds, and the final product length depends on the specificity of the enzyme; all-*E* short-chain prenyltransferases make products of lengths C_10_–C_25_ [where C_10_ is geranyl diphosphate (GPP), C_15_ is farnesyl diphosphate (FPP), C_20_ is geranyl geranyl diphosphate (GGPP) *etc*.], while longer C_30_–C_50_ products are made by medium-chain or long-chain all-*E*-prenyltransferases (Wang & Ohnuma, 2000 ▸; Ohnuma *et al.*, 1996 ▸).

Two divalent-metal-binding aspartic acid-rich DD*xx*D motifs termed the FARM and SARM (first and second aspartic acid-rich DD*xx*D motifs) are important in all-*E*-prenyltransferase mechanisms (Park *et al.*, 2012 ▸; Christianson, 2017 ▸; Chang *et al.*, 2021 ▸). In head-to-tail condensations, the FARM and SARM sites collectively bind three metal ions (two to FARM and a third to the SARM), facilitating co­ordination of the diphosphate (PP) moiety of the allylic substrate (DMAPP or FPP/GPP *etc.*), which will be ionized (the head). The metal groups, coordinating to both the enzyme and substrate, are believed to both facilitate ionization/cation formation and retain the cleaved diphosphate in position for the later elimination steps (Christianson, 2017 ▸; Liang, 2009 ▸). In addition to the metals, further DMAPP–protein inter­actions are made through basic residues such as Arg and Lys (Chang *et al.*, 2021 ▸; Park *et al.*, 2012 ▸). Binding of the allylic substrate to its binding site is thought to trigger a change in the enzyme from an open to a relatively closed conformation, helping to form the IPP binding site (the tail). Subsequent binding of IPP then facilitates the closure of the last four residues in the C-terminal tail over the active site (Park *et al.*, 2012 ▸, 2017 ▸). Active-site closure is important to the formation and protection of reactive species such as the allylic carbocation during condensation (Christianson, 2017 ▸). However, the closed active site must then open to allow release of the PP_i_ leaving group from the allylic/DMAPP site and to allow the elongated product to either leave the IPP site or be transferred back to the allylic/DMAPP site ready for another round of elongation (Park *et al.*, 2017 ▸).

Rv2173 has been identified as one of five *E*-isoprenyl diphosphate synthases (Rv0989c, Rv2173, Rv3383c, Rv0562 and Rv3398c) within *Mycobacterium tuberculosis* (Mann *et al.*, 2011 ▸). Each enzyme can accommodate different starting allylic substrates and produce different length products. It had been suggested that Rv0989c, with its unusual FARM and SARM motifs, is the main *M. tuberculosis* GPP (C_10_) synthase that uses DMAPP as a substrate (Mann *et al.*, 2011 ▸; Nagel *et al.*, 2018 ▸). Meanwhile, Rv0562 accepts a GPP starting substrate and produces a longer chain (C_45_) product that is likely to be used in menaquinone biosynthesis (Abe *et al.*, 2020 ▸). Rv3398c has been annotated as a FPP (C_15_) synthase that is limited to accepting GPP substrates (Dhiman *et al.*, 2004 ▸), while Rv3383c is a GPP (C_20_) synthase that is limited to accepting FPP substrates (Mann *et al.*, 2012 ▸). Functional studies have suggested that Rv2173 is versatile, capable of producing variable chain-length products from DMAPP, including products as long as GGPP (C_20_), although the enzymatic activity was episodic (Mann *et al.*, 2012 ▸). Product length determination is guided by the FARM and SARM motifs (Nagel *et al.*, 2018 ▸), with the size of the fifth residue before the FARM being associated with determining the chain length and larger residues limiting the size of the binding pocket (Ohnuma *et al.*, 1996 ▸). For Rv2173, this fifth residue is a tryptophan, suggesting that Rv2173 will produce a short-chain product. The exact functional role of Rv2173 in the cell is uncertain, although recent research appears to confirm a shorter product length, annotating Rv2173 as an *E*-C_10–15_ synthase and giving it a newly proposed biological function in the biosynthesis of glycosyl carrier lipids (Abe *et al.*, 2020 ▸). This annotation is hinted at by its genomic location immediately downstream of a putative α(1→6)-mannopyranosyltransferase (MptA, Rv2174) involved in lipomannan biosynthesis (Mishra *et al.*, 2011 ▸). This ability of Rv2173 to produce shorter products, including GPP, is also purported to facilitate functional redundancy within the bacterium, explaining for example why Rv0989c is not essential in knockout studies (Abe *et al.*, 2020 ▸).

## 2. Materials and methods

### 2.1. Macromolecule production

*M. tuberculosis* Rv2173 was expressed using a pDEST-17-based plasmid vector obtained from Dr Mann and coworkers containing an N-terminal 6×His tag (Table 1 ▸; Mann *et al.*, 2011 ▸, 2012 ▸). Purified pDEST-17-Rv2173 plasmid was transformed into chemically competent *Escherichia coli* BL21(DE3) or C41(DE3) cells for protein expression, with 100 µg ml^−1^ ampicillin used for selection (Table 1 ▸). Small overnight cultures (5 ml) in MDG medium (Studier, 2005 ▸) were used to inoculate 750 ml cultures in ZYM-5052 autoinduction medium (Studier, 2005 ▸). The cultures were grown at 37°C for 3 h followed by 18°C for ∼16 h, with shaking at 300 rev min^−1^. Other batches were grown using fermentation; a 100 ml overnight culture in MDG medium was used to inoculate 10 l ZYM-5052 medium in a 19.5 l fermenter (New Brunswick Scientific). The fermentative cultures were grown similarly to the flask-based cultures. The cells were harvested by centrifugation at 5000*g* for 30 min at 4°C and the cell pellets were stored at −80°C until use.

The cell pellets were resuspended in lysis buffer [for the apo and IPP crystals this was 50 m*M* Tris–HCl pH 7.0, 150 m*M* NaCl, 5%(*v*/*v*) glycerol and either 1 m*M* tris(2-carboxyethyl)phosphine (TCEP) or 1 m*M* β-mercaptoethanol; for the DMAPP-bound crystals the buffer composition was 50 m*M* HEPES pH 8.0, 5 m*M* MgCl_2_, 500 m*M* NaCl, 20 m*M* imidazole, 1 m*M* TCEP] containing a cOmplete Mini EDTA-free protease-inhibitor cocktail tablet. The resuspended cells were lysed by cell disruption using a Microfluidics cell disruptor at 124 MPa (Newton, USA). Following clarification (20 000*g*, 30 min, 4°C), the supernatant liquid was filtered (0.45 µm and then 0.22 µm) and purified by immobilized-metal affinity chromatography (IMAC) using a 5 ml HisTrap HP column (Cytiva). Rv2173 was eluted from the column using a gradient of elution buffer (the same as lysis buffer but containing 500 m*M* imidazole). For the protein used to create the apo and IPP crystals, further purification was performed by size-exclusion chromatography using a Superdex 200 10/300 GL column [50 m*M* Tris pH 7, 150 m*M* NaCl, 5%(*v*/*v*) glycerol]. Purified protein was stored at 4°C, or for longer-term storage at −80°C, until use.

### 2.2. Crystallization

Due to limited solubility, the tagged Rv2173 could only be concentrated to 0.7–2.2 mg ml^−1^. Crystallization-condition screening was carried out using a Cartesian robotic system with a 480-component screen (Moreland *et al.*, 2005 ▸) and the commercial MORPHEUS screen (Gorrec, 2009 ▸). Optimization of the most promising crystallization conditions was performed in 24-well hanging-drop plates, with successful crystallization in 1:1, 2:1 and 3:1 protein:reservoir solution ratios. IPP-bound and DMAPP-bound crystals were grown from co-crystallization experiments with the substrate (5–9 m*M*) added to the protein solution immediately prior to crystallization (Table 2 ▸). The crystals were harvested and then either immediately flash-cooled in liquid nitrogen (apo and DMAPP crystals) or soaked in cryoprotectant [well solution plus 25%(*v*/*v*) glycerol] and then flash-cooled.

### 2.3. Data collection and processing

Diffraction data were collected on the MX2 macromolecular crystallography beamline at the Australian Synchrotron (Aragão *et al.*, 2018 ▸) equipped with an ADSC Quantum 315r CCD detector (Meyer *et al.*, 2014 ▸). All data sets were processed and scaled with *XDS* (Kabsch, 2010*a* ▸,*b* ▸) and merged with *AIMLESS* (Evans & Murshudov, 2013 ▸). The most likely space group, *P*2_1_2_1_2 or *I*222, was determined using *POINTLESS* from the *CCP*4 suite (Agirre *et al.*, 2023 ▸). An *R*_free_ set corresponding to 5% of reflections was assigned. In the case of the apo crystal diffraction data, the twofold axis is along the shortest cell edge, which differs from the conventional assignment of the unit cell for *P*222 space groups and thus required swapping of the unit-cell axes. Data-processing statistics for the three PDB-deposited structures are reported in Table 3 ▸.

### 2.4. Structure solution and refinement

Matthews analysis (Matthews, 1968 ▸; Kantardjieff & Rupp, 2003 ▸) determined that there were likely to be two molecules in the asymmetric unit for *P*2_1_2_1_2 structures and one for *I*222. Molecular replacement was completed using *Phaser* (McCoy *et al.*, 2007 ▸) using initial refined but unpublished Rv2173 models. After molecular replacement, iterative rounds of refinement in *REFMAC*5 (Vagin *et al.*, 2004 ▸) in *CCP*4 (Agirre *et al.*, 2023 ▸) or *Phenix* (Liebschner *et al.*, 2019 ▸) were interspersed with rounds of model building in *Coot* (Emsley & Cowtan, 2004 ▸; Emsley *et al.*, 2010 ▸). Modelling of the metals as Ca^2+^ ions, based on the crystallization conditions, gave a better fit, minimizing the residual density compared with modelling as Mg^2+^, with similar *B* factors to those of other ligands. While Mg^2+^ is the assumed physiological ligand, accommodation of another metal is not unprecedented; Rv2173 activity has been tested by other groups with Mg^2+^, Mn^2+^ and Co^2+^, and it has been shown to be active with a similar product distribution (Abe *et al.*, 2020 ▸). In addition to the co-crystallized substrate components, several ligands (components of the crystallization condition or cryoprotectant conditions) were also modelled where appropriate. The refined final structures (Table 4 ▸) were deposited in the PDB as entries 8f8f, 8f8k and 8f8l.

During the building and refinement of the initial unpublished models (see the supporting information for further details) generated from molecular replacement with lower resolution data using a *BALBES*-modified (Keegan *et al.*, 2011 ▸; Long *et al.*, 2008 ▸) version of a geranylgeranyl pyrophosphate synthase from *Corynebacterium glutamicum* (PDB entry 3lk5; New York SGX Research Center for Structural Genomics, unpublished work; Supplementary Fig. S3), analysis using *Zanuda* (Lebedev & Isupov, 2014 ▸) helped to determine the correct axis for the twofold-symmetry element in the *P*2_1_2_1_2 space group.

### 2.5. Native mass spectrometry

Nano-electrospray ionization tips, with an orifice diameter of approximately 1 µm, were fabricated from 1.0 mm outer diameter and 0.75 mm inner diameter thin-walled glass capillaries (World Precision Instruments) using a P-2000 Micropipette Puller (Sutter). Pulled tips were gold-coated using a Q150R Plus Rotary Pumped Coater (Quorum). Prior to analysis, the protein sample (0.2 mg ml^−1^) was buffer-exchanged into 0.75 *M* ammonium acetate using Micro Bio-Spin Chromatography Columns (Bio-Rad) and then centrifuged at 17 000*g* for 2 min before dispensing into an electrospray capillary. Experiments were performed on a Synapt XS High Resolution Mass Spectrometer. The capillary voltage was typically 0.8 kV and the instrument was tuned for the transmission and preservation of high-mass protein complexes.

## 3. Results and discussion

### 3.1. Rv2173 is a homodimer

Consistent with most *E*-prenyltransferases, which exist as homodimers or heterodimers (Chang *et al.*, 2021 ▸), Rv2173 is a dimer in the crystal structure, with this biological assembly supported by native mass spectrometry (Supplementary Fig. S1). The apo structure was solved with a dimer in the asymmetric unit in space group *P*2_1_2_1_2, while both substrate-bound structures were solved in space group *I*222 with monomers in the asymmetric unit, generating dimers on the application of crystallographic symmetry. *PISA* analysis shows that the interface buries ∼15% of solvent-accessible surface area with a CCS score of 0.752 (Krissinel & Henrick, 2007 ▸). The monomeric units of each homodimer are oriented in a parallel fashion, with the lid helices located on the same side, such that both active sites open on the same face (Fig. 1 ▸*c*). This is a typical arrangement for homodimeric *E*-prenyltransferases, and it appears that dimer formation may be important in some of these enzymes for function via the creation of networks influencing the stability of substrate-binding sites (Chang *et al.*, 2013 ▸), while in others it may influence product cavity size (Artz *et al.*, 2011 ▸).

### 3.2. Rv2173 adopts a typical class I diterpene synthase fold

Rv2173 is folded as a decorated eight-helix bundle (Fig. 1 ▸*b*), typical of the all-α-helical class I diterpene synthase fold (Supplementary Figs. S2–S4; Christianson, 2017 ▸). Additional helices decorate the core fold to form a lid at the top of the active site and extensions involved in dimer interactions (Figs. 1 ▸*b* and 1 ▸*c*, Supplementary Figs. S2–S4). These lid helices have been implicated in controlled opening and closure of the active site during catalytic cycling, protecting the reactive species generated and allowing substrate binding/exchange and product exit (Sun *et al.*, 2005 ▸; Park *et al.*, 2012 ▸). The FARM and SARM motifs face each other from opposite helices of the substrate-binding cleft (Fig. 1 ▸*b*; Supplementary Figs. S2–S4; Christianson, 2017 ▸). All three structures are very similar (0.36–0.65 Å r.m.s.d. across all C^α^ atoms as calculated by *SSM*; Krissinel & Henrick, 2004 ▸), with the main variation being in the completeness of the C-terminus; the apo structure is missing the last four residues and the DMAPP-bound structure the last eight residues, while the IPP-bound structure has a complete C-terminus. It is these C-terminal residues in conjunction with the aforementioned lid helices that are likely to close the active site fully during the reaction cycle.

### 3.3. Active-site and C-terminal closure

**Figure 2:**
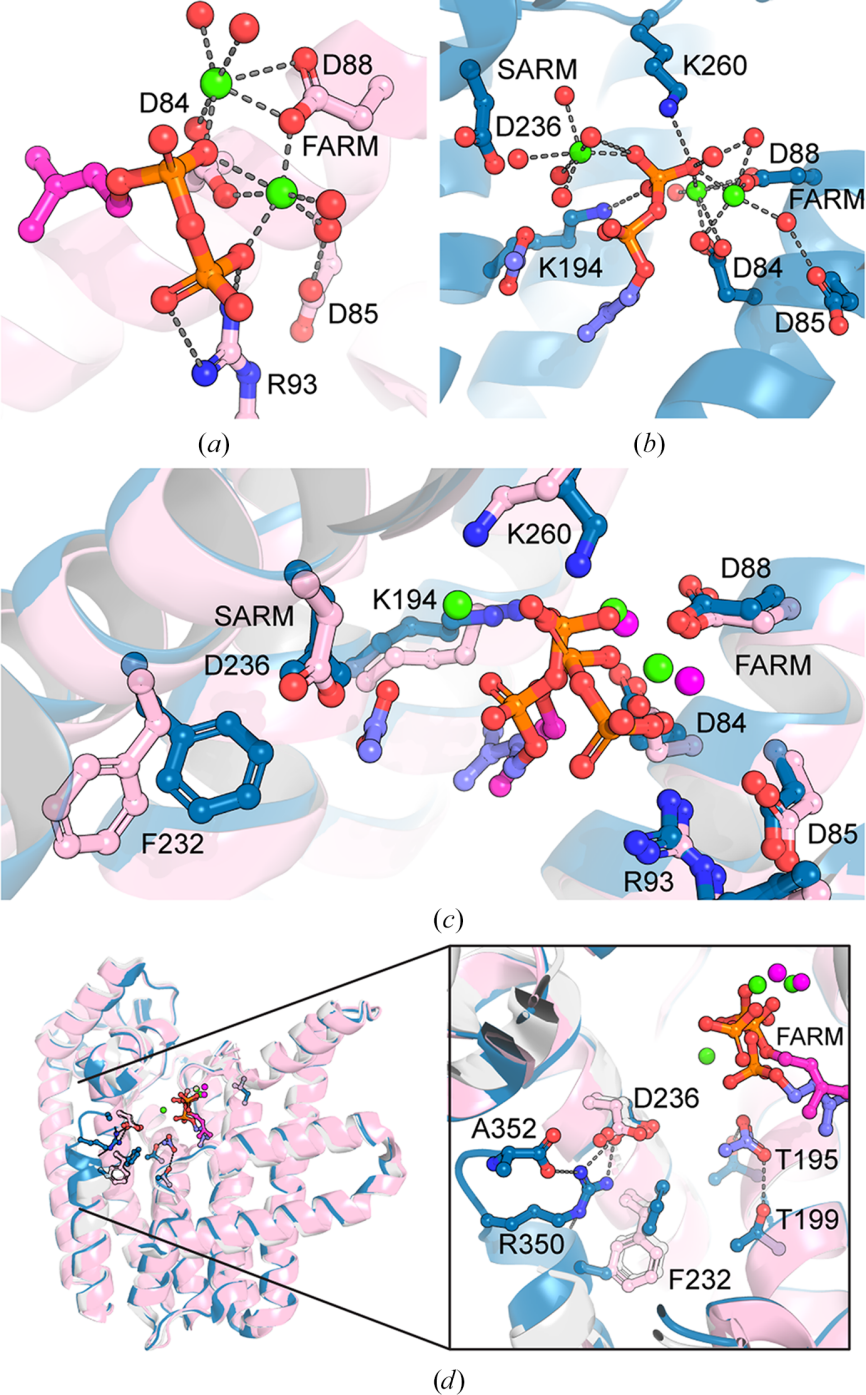
(*a*) DMAPP binding into FARM in Rv2173. (*b*) IPP binding into FARM and SARM in Rv2173. (*c*) Overlay of the FARM and SARM allylic/DMAPP binding site in DMAPP-bound and IPP-bound structures. The DMAPP-bound structure is coloured light pink, with metal ions as green spheres and DMAPP as pink sticks. The IPP-bound structure is coloured blue, with metal ions as green spheres and the IPP and acetate as purple sticks. Waters are shown as red spheres and polar contacts as grey dashes. (*d*) Overlay of the IPP-bound (blue), DMAPP-bound (light pink) and apo (white) structures with a focus on the differences in the C-terminus leading to active-site closure.

The IPP-bound structure appears to be the most closed form with a fully ordered C-terminus. It has three metal ions bound: two to the FARM unit (DDLID; residues 84–88) and one near the SARM unit (DDVLGVFGD; residues 236–244), with the IPP occupying the putative allylic/DMAPP site, coordinating all three metal ions and making interactions with Lys194 and Lys260 (Fig. 2 ▸*b*). While surprising, there is precedent for IPP binding to the allylic/DMAPP site in other *E*-prenyltransferases (Kavanagh, Dunford *et al.*, 2006 ▸; Kavanagh, Guo *et al.*, 2006 ▸; Guo *et al.*, 2007 ▸). Interestingly, the third metal ion does not make any direct interaction with the SARM Asp236 as might have been expected from other three-metal-bound *E*-prenyltransferase structures (for example PDB entry 4h5e; Park *et al.*, 2012 ▸). The significance of this variance in metal-ion placement seen in Rv2173 is uncertain, although it has been suggested all three metal ions are needed for successful carbocation formation as well as for retention of the cleaved diphosphate for its role in elimination (Christianson, 2017 ▸). Variations in the FARM and SARM motifs have been proposed to influence how the metals coordinate (Artz *et al.*, 2011 ▸; Nagel *et al.*, 2018 ▸); however, it is important to note that IPP is not the physiological ligand for the metal-associated allylic binding site. Additionally, in all structures, and even in this most closed form, the basic residue Lys43, which is likely to be important for the binding of the PP group in the IPP site, is poorly ordered and this disorder may also influence variability in positioning of the metal ions.

The DMAPP-bound structure represents the most open form, with no electron density observed for the last eight residues from the C-terminus. Two metal ions bind to the FARM, coordinating to the DMAPP bound in the allylic/DMAPP site (Fig. 2 ▸*a*). Notably, in other structural studies of related enzymes it is common for not all three metal ions to be observed (Guo *et al.*, 2007 ▸). Compared with IPP, the DMAPP diphosphate (PP) group appears to exhibit greater coordination to both FARM-site metal ions present, whereas in the IPP-bound structure IPP appears to be coordinated by just one of its phosphate groups (Figs. 2 ▸*a*–2 ▸*c*). This difference is consistent with the observation that the positioning of IPP and DMAPP in this site differs slightly (Fig. 2 ▸*c*), with the binding mode observed for DMAPP best mirroring other complexes (for example various GGPP synthase complexes; Guo *et al.*, 2007 ▸).

Comparison to related enzyme structures with IPP sites occupied (for example PDB entries 2e8v, 2e8t and 2e8u; Guo *et al.*, 2007 ▸) has helped to identify the IPP binding site in Rv2173 (Lys43, Arg90, His77, Arg46, Thr195 and Phe232). While this putative binding site is never observed to be occupied by IPP, in the case of the IPP-bound structure an acetate artefact from the crystallization condition appears to occupy part of this site, although the site itself (for example Lys43) shows aspects of disorder consistent with its lack of substrate occupancy (Fig. 2 ▸*d*). We observe a role for Phe232 from this site in changes associated with C-terminal closure in the IPP-bound structure. In this structure, Arg350 faces into the active site and forms hydrogen-bonding interactions with the final C-terminal residue Ala352, as well as Asp236 from the SARM motif and the backbone of Phe232 (Fig. 2 ▸*d*). Compared with the apo and DMAPP-bound structures, there is notable movement of Phe232, the side chain of which moves inwards to face the active site, facilitating its closure (Fig. 2 ▸*d*). Studies of closure mechanisms for FPP synthase (FPPS) propose a cascade of changes from IPP occupation to C-terminal closure (Park *et al.*, 2012 ▸). Although limited conservation suggests that this mechanism is unlikely to be fully preserved in Rv2173, these three residues (Arg350/FPPSArg351; Phe232/FPPSPhe239; Asp236/FPPSAsp243) are conserved, correlating with their potential shared importance.

### 3.4. Chain length of product

**Figure 3:**
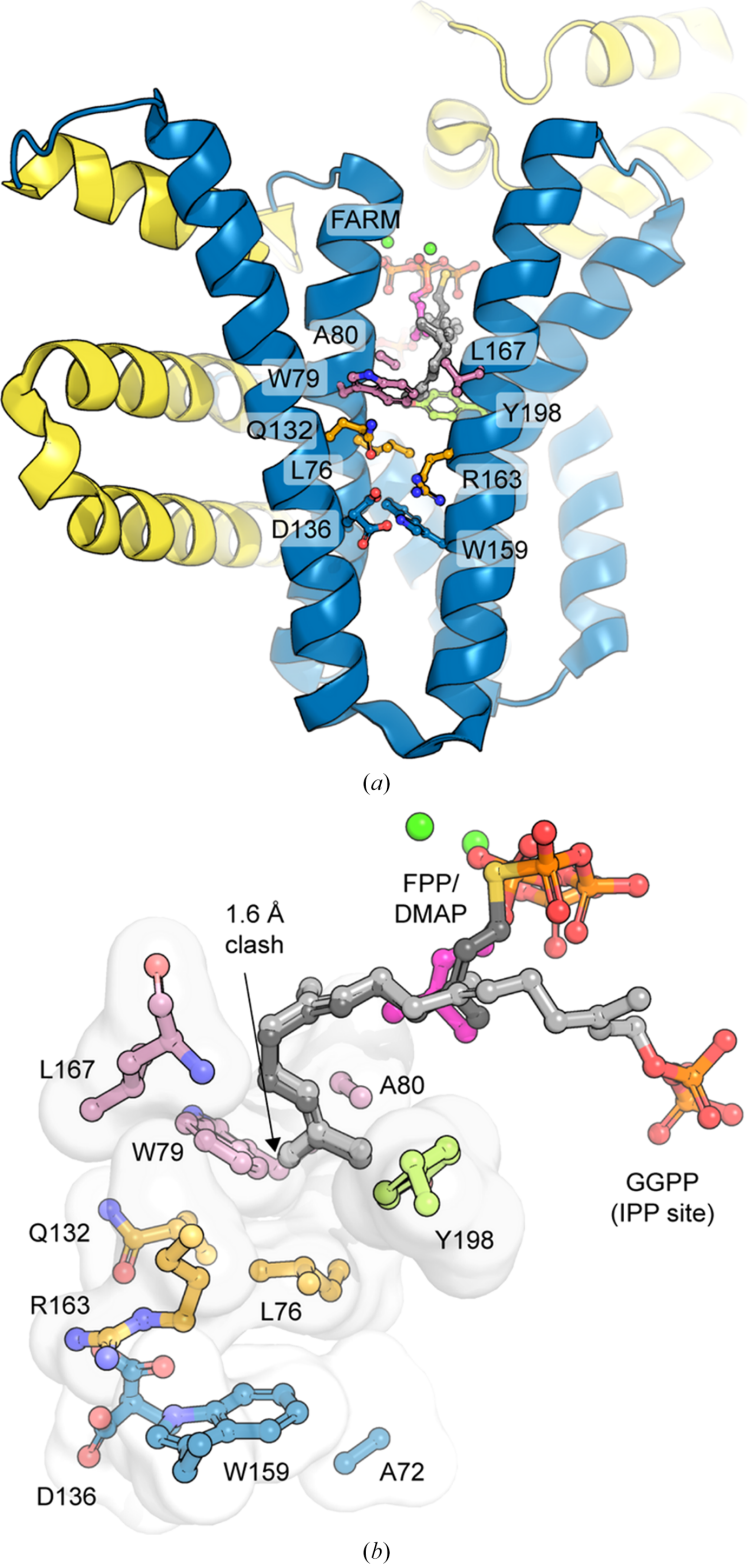
Product-length pocket and floors. (*a*) The three floors in Rv2173: floor 1 in light pink, floor 2 in orange and floor 3 in blue with Tyr198 in lime. (*b*) Close-up showing the floor residues as a surface. In both panels to mark the allylic DMAPP site, DMAPP is shown as magenta sticks, with metal ions as green spheres. The FPP (C_15_) bound in as the substrate in this site from a superposition with PDB entry 2e8t is shown in dark grey. The GGPP (C_20_) from PDB entry 2e8v as the product in the IPP site is shown as light grey sticks. GGPP comes into close contact with the floor residues, suggesting that a smaller size (C_10_ or C_15_) product would be preferred.

Prior functional studies on Rv2173 have been inconsistent, with some evidence that Rv2173 can, episodically at least, produce products as long as GGPP (C_20_; Mann *et al.*, 2012 ▸), although more recent research appears to suggest a shorter principal product, annotating Rv2173 as an *E*-C_10–15_ synthase (Abe *et al.*, 2020 ▸). We performed various soaking and co-crystallization experiments with potential substrates/products longer than C_5_, but no associated ligand density was observed. We therefore turned to analysis of the structure and sequence of Rv2173 to give insight into the most plausible maximal product length. The fifth residue pre-FARM in IPP synthases helps to determine the product length (Ohnuma *et al.*, 1996 ▸; Feng *et al.*, 2020 ▸). In Rv2173, this residue is a large bulky tryptophan (Trp79), suggesting relatively short products. However, the product-length determination can be more nuanced than a single residue, with some suggestions for CrtE (PDB entry 6sxl) that three ‘floors’ of residues in the substrate-binding cleft are influential in determining product length; bulky residues in floor 1 limit the cavity size to a C_15_/FPP product, while access past this floor with bulky residues in floors 2 or 3 result in C_20_/GGPP and C_25_/GFPP products, respectively (Feng *et al.*, 2020 ▸). The residues equivalent to floor 1 in Rv2173 are Trp79, Ala80 and Leu167, those equivalent to floor 2 are Gln132, Leu76 and Arg163, and those equivalent to floor 3 are Trp159, Ala72 and Asp136 (Fig. 3 ▸), supporting the idea that Rv2173 produces shorter length products such as FPP. This proposition is further supported by comparison of Rv2173 with a GGPP synthase structure with longer chain substrate/product analogues (for example PDB entries 2e8v and 2e8t; Guo *et al.*, 2007 ▸), which appears to confirm that Trp79 and Leu167 will limit the pocket size, and another bulky residue, Tyr198, will block access to the bottom of the pocket (Fig. 3 ▸). Together, these factors suggest that Rv2173 could best accommodate a final substrate the size of GPP, yielding a product the size of FPP and supporting the later functional annotation as a C_10_–C_15_ product-length synthase.

## 4. Conclusion

Rv2173 is a homodimer with a canonical class I diterpene synthase fold, with structural analysis identifying some conserved mechanistic features in C-terminal closure and supporting its annotation as a short-chain (C_10_–C_15_) product-length synthase.

## 5. Related literature

The following references are cited in the supporting information for this article: Bricogne *et al.* (2017 ▸), Evans (2006 ▸), Madeira *et al.* (2024 ▸) and Robert & Gouet (2014 ▸).

## Supplementary Material

PDB reference: Rv2173 from *M. tuberculosis*, apo form, 8f8f

PDB reference: with IPP bound, 8f8k

PDB reference: with DMAP bound, 8f8l

Supplementary Figures and supporting information for Section 2.4. DOI: 10.1107/S2053230X25002298/us5157sup1.pdf

## Figures and Tables

**Table 1:** Macromolecule-production information

Source organism	*Mycobacterium tuberculosis*
DNA source	Genomic DNA
Expression vector	pDEST-17
Expression host	*Escherichia coli* BL21(DE3) and C41(DE3)
Complete amino-acid sequence of the construct produced	MSYYHHHHHHLESTSLYKKAGSAAALFRFKKEPFTMAGAITDQLRRYLHGRRRAAAHMGSDYDGLIADLEDFVLGGGKRLRPLFAYWGWHAVASREPDPDVLLLFSALELLHAWALVHDDLIDRSATRRGRPTAQLRYAALHRDRDWRGSPDQFGMSAAILLGDLAQVWADDIVSKVCQSALAPDAQRRVHRVWADIRNEVLGGQYLDIVAEASAAESIESAMNVATLKTACYTVSRPLQLGTAAAADRSDVAAIFEHFGADLGVAFQLRDDVLGVFGDPAVTGKPSGDDLKSGKRTVLVAEAVELADRSDPLAAKLLRTSIGTRLTDAQVRELRTVIEAVGARAAAESRIAALTQRALATLASAPINATAKAGLSELAMMAANRSA

**Table 2:** Crystallization

	Apo	IPP-bound	DMAP-bound
Method	Vapour diffusion		
Plate type	96-well sitting drop and 24-well hanging drop		
Temperature (K)	291		
Protein concentration (mg ml^−1^)	0.7–2.2		
Buffer composition of protein solution	50 m*M* Tris pH 7, 150 m*M* NaCl, 5%(*v*/*v*) glycerol, 1 m*M* TCEP or β-mercaptoethanol	50 m*M* Tris pH 7, 150 m*M* NaCl, 5%(*v*/*v*) glycerol, 1 m*M* TCEP or β-mercaptoethanol, 5 m*M* IPP	50 m*M* HEPES pH 8.0, 5 m*M* MgCl_2_, 500 m*M* NaCl, 200–250 m*M* imidazole, 1 m*M* TCEP, 9 m*M* DMAPP
Composition of reservoir solution	0.1 *M* Bicine–Tris pH 8.5, 10% PEG 4000, 24% glycerol, 0.03 *M* DC mix CaCl_2_ and MgCl_2_	0.1 *M* Tris pH 7.5, 0.2 *M* calcium acetate, 20% PEG 3350	0.1 *M* Bicine–Tris pH 8.3, 12.5% PEG 4K, 25% glycerol, 0.03 *M* CA mix CaCl_2_ and MgCl_2_
Volume and ratio of drop	1–4 µl total volume; protein:well ratios 1:1, 2:1, 3:1		
Volume of reservoir (µl)	500–600		

**Table 3:** Data collection and processing Values in parentheses are for the outer resolution shell.

Structure	Apo	IPP-bound	DMAPP-bound
PDB code	8f8f	8f8k	8f8l
Diffraction source	MX2 beamline, Australian Synchrotron	MX2 beamline, Australian Synchrotron	MX2 beamline, Australian Synchrotron
Wavelength (Å)	0.953700	0.953700	0.953700
Space group	*P*2_1_2_1_2	*I*222	*I*222
*a*, *b*, *c* (Å)	81.80, 190.71, 66.48	65.66, 82.69, 189.38	66.54, 83.84, 188.12
α, β, γ (°)	90, 90, 90	90, 90, 90	90, 90, 90
Resolution range (Å)	19.76–2.00 (2.00–2.04)	19.80–2.20 (2.20–2.27)	19.58–2.20 (2.20–2.27)
No. of unique reflections	71090 (4495)	26593 (2255)	26527 (2016)
No. of observations	103648 (66769)	389059 (33239)	365883 (18434)
Multiplicity	14.6 (14.9)	14.6 (14.7)	13.8 (9.1)
*R*_merge_ (all *I*−/*I*+)	0.124 (3.25)	0.187 (4.175)	0.229 (2.696)
*R*_p.i.m._ (all *I*+/*I*−)	0.033 (0.865)	0.051 (1.124)	0.064 (0.923)
CC_1/2_	0.999 (0.384)	0.999 (0.359)	0.996 (0.238)
〈*I*/σ(*I*)〉†	17.8 (1.2)	15.4 (0.9)	12.6 (0.9)
Completeness (%)	99.9 (100)	99.9 (100)	97.2 (82.0)
Wilson *B* factor (Å^2^)	38.84	45.52	35.71

† The mean *I*/σ(*I*) in the outer shell falls below 2.0 at resolutions above 2.11, 2.44 and 2.44 Å for the apo, IPP and DMAPP data sets, respectively. Analyses of merged CC_1/2_ correlations between intensity estimates from half-data sets (Karplus & Diederichs, 2015 ▸) were used to influence the high-resolution cutoff for data processing.

**Table 4:** Structure refinement

Structure	Apo	IPP-bound	DMAPP-bound
PDB code	8f8f	8f8k	8f8l
Resolution range (Å)	19.76–2.00 (2.00–2.03)	19.80–2.20 (2.20–2.28)	19.58–2.20 (2.20–2.28)
Completeness (%)	99.98	99.94	97.22
σ Cutoff	1.34	1.34	1.34
No. of reflections			
Working set	67437 (2520)	25242 (2448)	25173 (2059)
Test set	3577 (140)	1335 (148)	1336 (128)
*R*_work_	0.2121 (0.3807)	0.2083 (0.3782)	0.2108 (0.3213)
*R*_free_	0.2384 (0.4367)	0.2388 (0.4013)	0.2436 (0.3300)
No. of non-H atoms			
Total	5642	2806	2768
Protein	5318	2668	2608
Ligand	55	46	22
Solvent	269	92	138
R.m.s. deviations			
Bond lengths (Å)	0.002	0.002	0.002
Angles (°)	0.454	0.488	0.377
Average *B* factors (Å^2^)			
Protein	47.00	55.30	43.54
Ligand	65.57	61.39	50.17
Solvent	48.66	53.92	41.91
Ramachandran plot			
Most favoured (%)	99.42	98.29	99.42
Allowed (%)	0.58	1.71	0.58
