# Supplementary material for: Structures of Mycobacterium tuberculosis isoprenyl diphosphate synthase Rv2173 in substrate-bound forms
Source: Acta Crystallogr F Struct Biol Commun. 2025 Apr 1;81(Pt 5):193–200. doi: 10.1107/S2053230X25002298 (PMC12035560; doi:10.1107/S2053230X25002298)
Supplement: Supplementary file 1 [file f-81-00193-sup1.pdf]

STRUCTURAL BIOLOGY
COMMUNICATIONS

Volume 81 (2025)

Supporting information for article:

Structures of *Mycobacterium tuberculosis* Rv2173 isoprenyl diphosphate synthase in substrate-bound forms

James A. Titterington, Ngoc Anh Thu Ho, Charles P. H. Beasley, Francis Mann, Edward N. Baker, Timothy M. Allison and Jodie M. Johnston

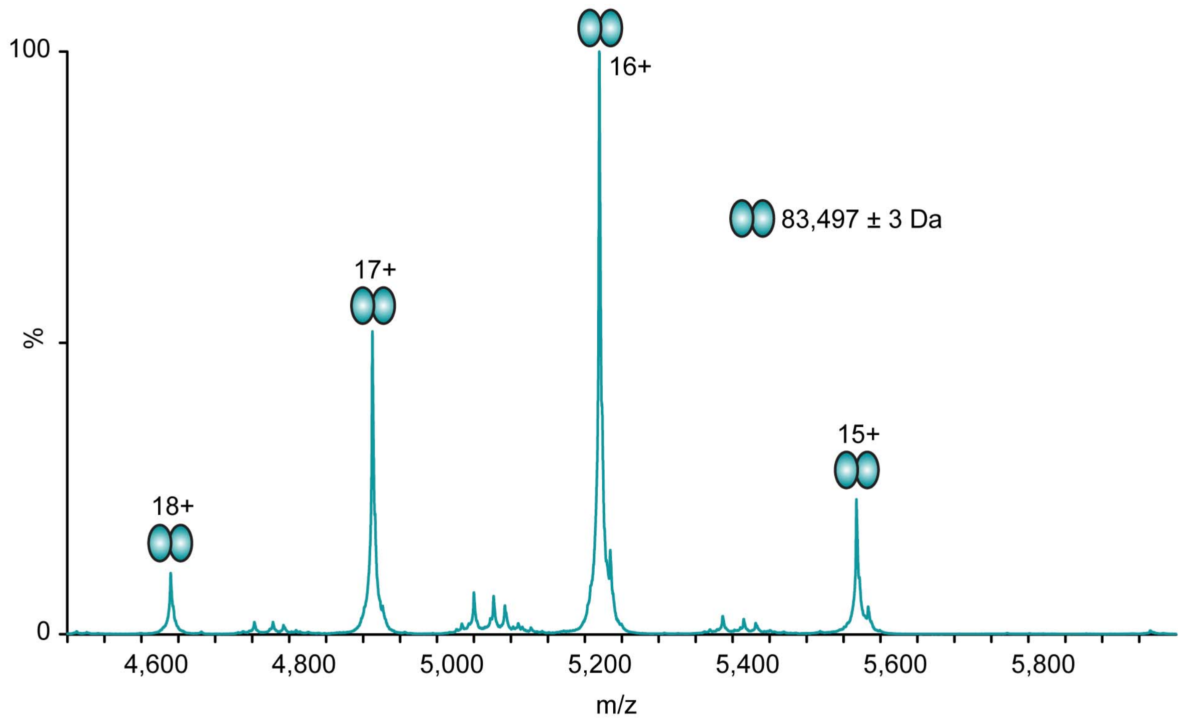

Figure S1 Native mass spectrum of Rv2173. The spectrum was recorded of 0.2 mg/mL Rv2173 exchanged into 750 mM ammonium acetate, using a Synapt XS mass spectrometer modified with a 32,000 m/z quadrupole and tuned for the transmission of large protein complexes, using offline nano-electrospray ionisation.

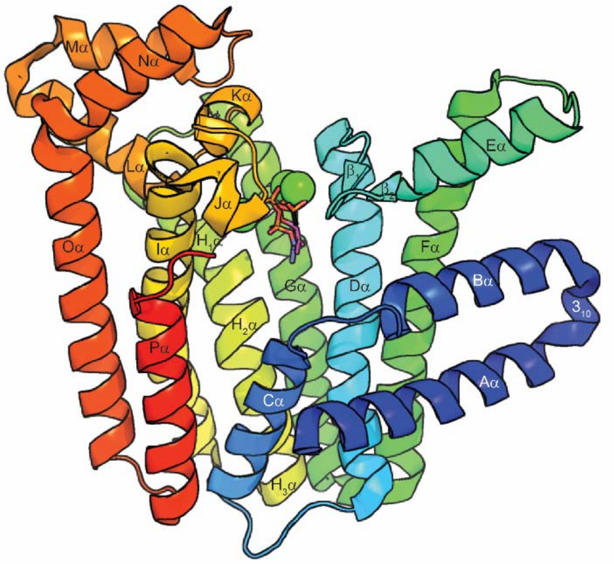

Figure S2 Monomer structure of Rv2173 as a chainbow with secondary structures labelled (to match sequence alignments in S3 and S3). N-terminal helices comprise Aα-Bα; core eight α-helices comprise Cα-Dα, Fα-Iα and Oα-Pα and the lid helices (and sheet) comprise Eα, β_1-β_2, and Jα-Nα. The N-terminal helices and lid helices (of some sort) are a common feature of the fold, though the lid helices can be variable.

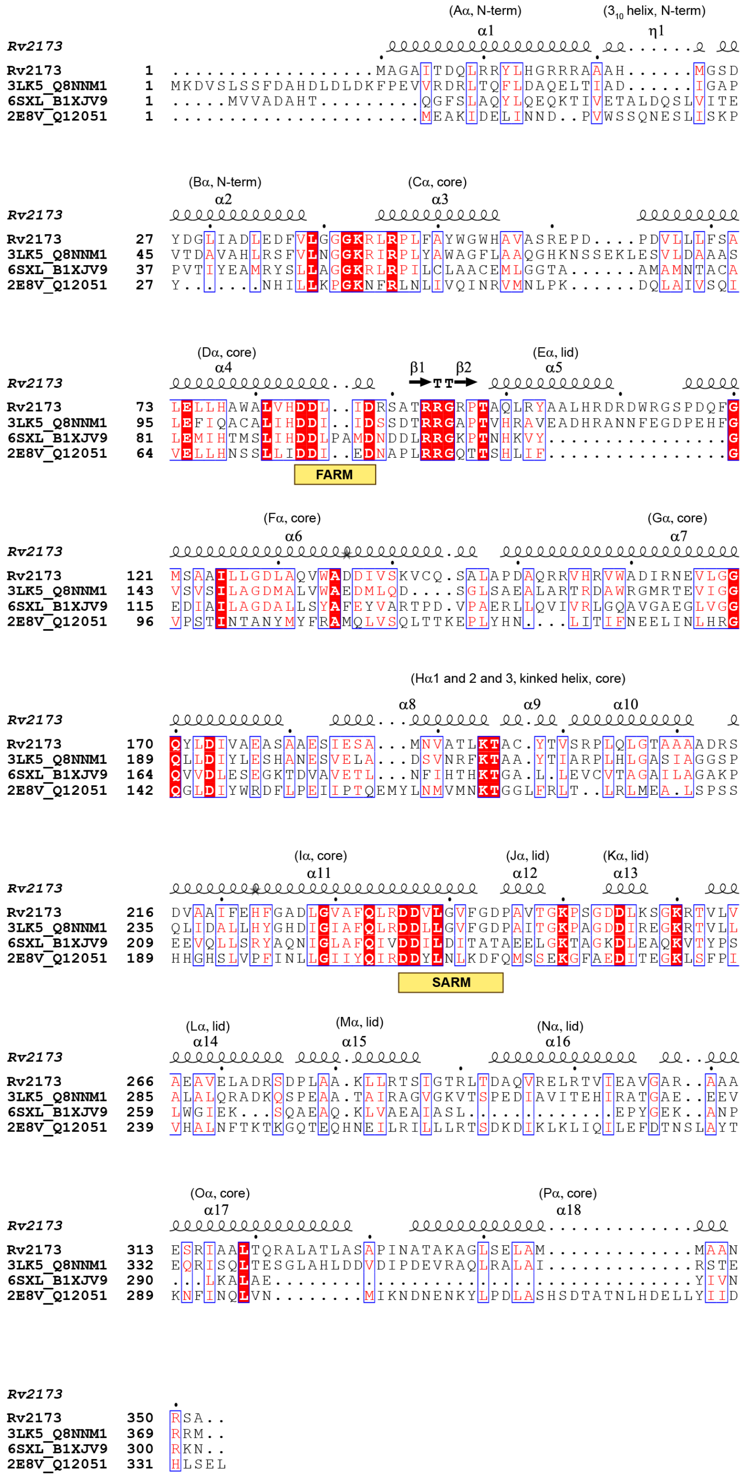

Figure S3 Clustal Omega (Madeira *et al.*, 2024) and ESPRIPT (Robert & Gouet, 2014) sequence alignment of the Rv2173 sequence with the UniProt sequences of three structures referred to in the main text: 3LK5 UniProt ID Q8NNM1, *Corynebacterium glutamicum* geranylgeranyl pyrophosphate synthase which shares 42% amino acid sequence identity to Rv2173, and was the original molecular replacement model; 2E8V UniProt ID Q12051, representative of the yeast (*Saccharomyces cerevisiae*) geranylgeranyl pyrophosphate synthase structures discussed, and 6SXL UniProt ID

B1XJV9, a *Picosynechococcus sp.* geranylgeranyl pyrophosphate synthase, discussed in relation to product length determination. FARM and SARM are annotated in yellow boxes and the secondary structure as annotated in S2 is labelled above the sequence.

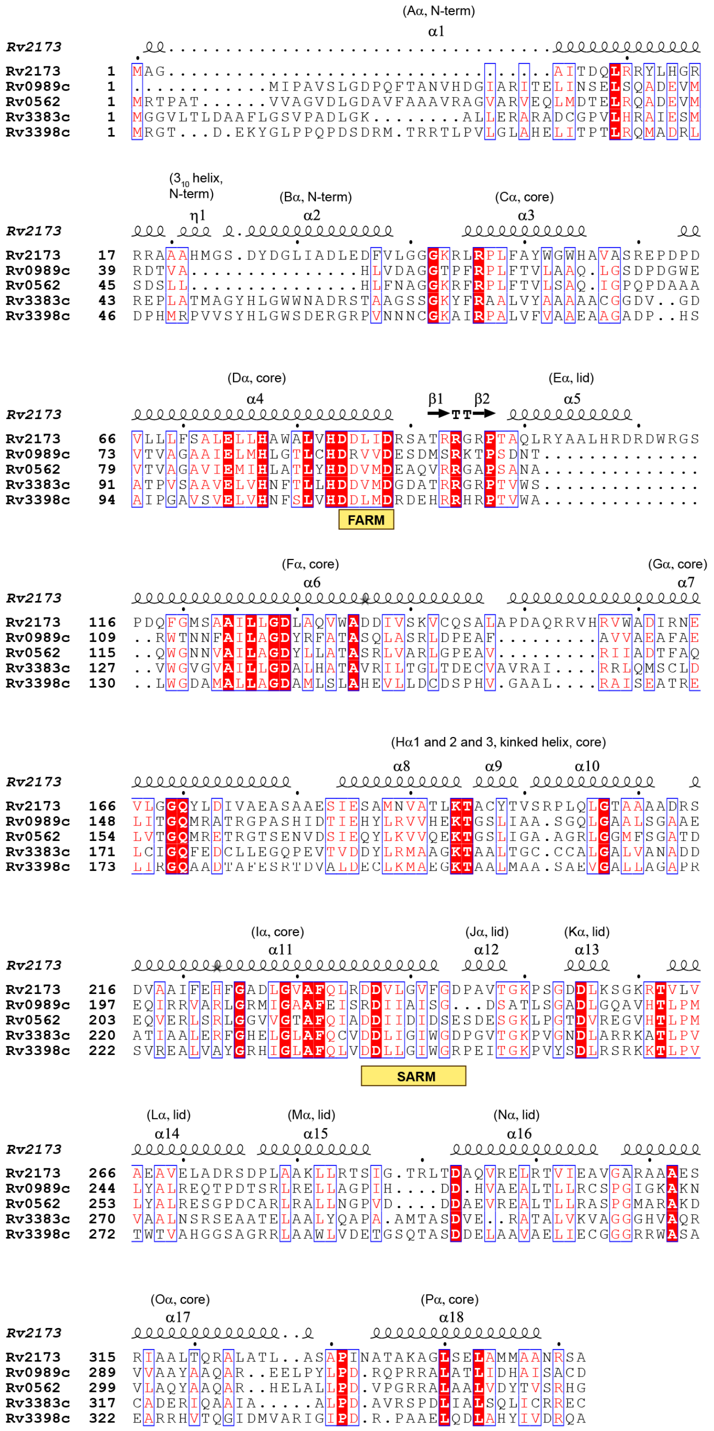

Figure S4 Clustal Omega (Madeira *et al.*, 2024) and ESPRIPT (Robert & Gouet, 2014) sequence alignment of the all *E*-isoprenyl diphosphate synthases in *Mycobacterium tuberculosis* including Rv2173. FARM and SARM are annotated in yellow boxes and the secondary structure as annotated in

Fig. S2 is labelled above the sequence. Note the unusual substitutions of Arg in the FARM and SARM of Rv0989c, thought to confer shortened product-length formation.

S1. Supporting information for section 2.4 detailing initial (unpublished) model generation, building, and refinement

Initial (unpublished) apo structures of Rv2173 served as good quality molecular replacement model for the structures reported in this paper. They were of lower resolution than the apo structure reported in the paper. The first of these models were solved via molecular replacement using a BALBES (Keegan *et al.*, 2011, Long *et al.*, 2008) generated template model, which was a modified version of the geranylgeranyl pyrophosphate synthase from *Corynebacterium glutamicum* (PDB code 3LK5) with the Rv2173 sequence docked onto it. Rv2173 and 3LK5 sequence have 42% amino acid sequence identity and an alignment is shown as part of Fig. S3. After molecular replacement the first model underwent multiple rounds of extensive manual model building using Coot (Emsley & Cowtan, 2004, Emsley *et al.*, 2010) iterated with refinement in Refmac5 (Vagin *et al.*, 2004) and the program BUSTER (Bricogne G., 2017) to generate the apo 2.6 Å initial model 1. It was also during this process that we undertook Zanuda (Lebedev & Isupov, 2014) analysis and cell-reindexing as mentioned in section 2.4. Subsequently, several better resolution apo datasets were collected and one of these (~2.2 Å) was used to generate initial model 2. The first model was used for molecular replacement with this data (with retention of the same R_{free} set during scala (Evans, 2006, Evans & Murshudov, 2013) reduction to reduce refinement bias), which again underwent a number of rounds of manual model building using Coot iterated with refinement in Refmac5 (Vagin *et al.*, 2004) and Buster (Bricogne G., 2017) to produce the improved initial model 2 (R 20.97/R_{free} 26.34%). The A chain of initial model 2 was used for subsequent molecular replacement searches with newer data.

References

Bricogne G., B. E., Brandl M., Flensburg C., Keller P., Paciorek W., Roversi P, Sharff A., Smart O.S., Vonrhein C., Womack T.O. (2017). *BUSTER version 2.10.0.*

Emsley, P. & Cowtan, K. (2004). *Acta crystallographica section D: biological crystallography* **60**, 2126-2132.

Emsley, P., Lohkamp, B., Scott, W. G. & Cowtan, K. (2010). *Acta Crystallogr D Biol Crystallogr* **66**, 486-501.

Evans, P. (2006). *Acta Crystallogr D Biol Crystallogr* **62**, 72-82.

Evans, P. R. & Murshudov, G. N. (2013). *Acta Crystallogr D Biol Crystallogr* **69**, 1204-1214.

Keegan, R. M., Long, F., Fazio, V. J., Winn, M. D., Murshudov, G. N. & Vagin, A. A. (2011). *Acta Crystallogr D Biol Crystallogr* **67**, 313-323.

Lebedev, A. A. & Isupov, M. N. (2014). *Acta Crystallogr D Biol Crystallogr* **70**, 2430-2443.

Long, F., Vagin, A. A., Young, P. & Murshudov, G. N. (2008). *Acta Crystallogr D Biol Crystallogr* **64**, 125-132.

Madeira, F., Madhusoodanan, N., Lee, J., Eusebi, A., Niewielska, A., Tivey, A. R. N., Lopez, R. & Butcher, S. (2024). *Nucleic Acids Res* **52**, W521-W525.

Robert, X. & Gouet, P. (2014). *Nucleic Acids Res* **42**, W320-324.

Vagin, A. A., Steiner, R. A., Lebedev, A. A., Potterton, L., McNicholas, S., Long, F. & Murshudov, G. N. (2004). *Acta Crystallographica Section D: Biological Crystallography* **60**, 2184-2195.
